# Supplementary material for: Relationships among the β3-adrenargic receptor gene Trp64Arg polymorphism, hypertension, and insulin resistance in a Japanese population
Source: PLoS One. 2021 Aug 4;16(8):e0255444. doi: 10.1371/journal.pone.0255444 (PMC8336805; doi:10.1371/journal.pone.0255444)
Supplement: Attachment — Submitted filename: Response to Reviewers.docx [file pone.0255444.s001.docx]

Dear Dr. Raffaella Buzzetti,

Thank you for the thoughtful and constructive feedback you provided regarding our manuscript, ‘Relationships among the β3-adrenargic receptor gene Trp64Arg polymorphism, hypertension, and insulin resistance in a Japanese population’. We have carefully reviewed the comments and revised the manuscript on the basis of the reviewers’ comments. Our point-by-point responses to the reviewers’ comments are listed below this letter. Changes to the manuscript are shown in red font in a separate file labeled 'Revised Manuscript with Track Changes'.

We hope that you find the current version of the manuscript suitable for publication. Thank you for your consideration. We look forward to the publication of our manuscript in the PLOS ONE.

Sincerely,

Youhei Yamada

Reviewer #1

1. Could the Authors specify if they used some exclusion criteria to enrol the subjects?

Response: The participants recruitment chart is shown in the newly created Figure 1.

2. It would be interesting if the Authors provide the images of genotyping results.

Response: We provide the images of genotyping results in the newly created Figure 2.

3. Did The Authors evaluate the sample size and the could they report the power of the study ?

Response: We didn't performed a power calculation before starting the study. After the fact, we confirmed the detection power to see if the sample size was appropriate, but sufficient detection power was confirmed.

4. All the data were normally distributed? Which kind of test they used to verify data normality?

Response: Not all the data were normally distributed. We used Shapiro-Wilk test to verify data normality. In this study, we addressed the problem of normality by using a highly robust analytical method.

5. The discussion section is limited, the Authors should add some sentence in which they explain the possible implication of their findings in the clinical practice.

Response: We increased the text of the discussion section and emphasized the significance of our research results.

6. The Authors should specific in material and method the range age of subjects enrolled in the study.

Response: In the present study, data on 1191 voluntary participants from 40 years of age who underwent the comprehensive health examination between March 2014 and January 2017 were available.

7. It would be interesting subdivide the subjects in two groups according gender, to evaluate possible differences with the polymorphisms.

Response: In response to the proposal, we newly analyzed by gender. The results of this study were found to be prominent in women.

8. In the study there are no information regarding the lipid profile of the subjects. Dyslipidaemia, comprising altered ratio of high TC level and isolated evaluation of the LDL or TG, is usually associated with increased blood pressure (BP) levels. These considerations should be take into account in the discussion section.

Response: We have shown the lipid profile in Tables 1, 2 and 3. Taking into account the lipid profile did not change the trends in our findings.

Reviewer #2

1. Have Authors perfomed a power calcualtion before starting the study? Could they report such a calculation?

Response: We didn't performed a power calculation before starting the study. After the fact, we confirmed the detection power to see if the sample size was appropriate, but sufficient detection power was confirmed.

2. The sentence “In the present study, the ADRB3 polymorphism did not correlate with

phenotypes (obesity, hypertension, and insulin resistance” should be removed from the abstract as it does not add anithing fundamental but,even complicates the comprehension of the text.

Response: I deleted the sentence you pointed out.

3. Please change HOMA-R in HOMA-IR as it is generally reported in the international abbreviation

Response: I changed the word you pointed out.

4. Please report the cut-off for defining Japanese population concerning the BMI ( normal, overweight, obese)

Response: We have shown in Table 1 the cutoff for Japanese BMI.

5. Other susceptible genes have been demostrated to be linked to obesity, insulin resistance and hypertension in caucasian population. Among all, adiponectin stands out (PMID: 17030959); please refer to that article and comment on the most important genes already demonstrated to be associated with insulin resistance obesity and hypertension in Japanese population. Surely, it would be very useful for readers

Response: We mentioned adiponectin in the discussion section with reference to the literature you provided.

6. Have Authors any informaton about the diabetes type which affected more less 10 percent of the investigated subjects?

Response: In our study, type 1 diabetes was excluded from the analysis using HOMAβ.

7. Exercise and drinking habits should be better quantified. Please comment on that.

Response: The lifestyle survey was evaluated qualitatively according to the questionnaire. We are very sorry, but we cannot use quantitative information about exercise and drinking for analysis.
